# Isovitexin Is a Direct Inhibitor of *Staphylococcus aureus* Coagulase

**DOI:** 10.4014/jmb.2105.05013

**Published:** 2021-08-19

**Authors:** Hua Xiang, Panpan Yang, Li Wang, Jiaxin Li, Tiedong Wang, Junze Xue, Dacheng Wang, Hongxia Ma

**Affiliations:** 1 College of Animal Medicine, Jilin Agricultural University, Changchun 130118, P.R. China; 2 College of Basic Medical Science, Jilin University, Changchun 130012, P.R. China; 3 College of Animal Science, Jilin University, Changchun 130062, P.R. China; 4 College of Animal Science and Technology, Jilin Agricultural University, Changchun 130118, P.R. China; 5 The Key Laboratory of New Veterinary Drug Research and Development of Jilin Province, Jilin Agricultural University, Changchun 130118, P.R. China; 6 College of Life Science, Jilin Agricultural University, Changchun 130118, P.R. China; 7 The Engineering Research Center of Bioreactor and Drug Development, Ministry of Education, Jilin Agricultural University, Changchun 130118, P.R. China

**Keywords:** Isovitexin, coagulase, pneumonia, *Staphylococcus aureus*, inhibitor

## Abstract

*Staphylococcus aureus* (*S. aureus*) is a major pathogen that causes human pneumonia, leading to significant morbidity and mortality. *S. aureus* coagulase (Coa) triggers the polymerization of fibrin by activating host prothrombin, which then converts fibrinogen to fibrin and contributes to *S. aureus* pathogenesis and persistent infection. In our research, we demonstrate that isovitexin, an active traditional Chinese medicine component, can inhibit the coagulase activity of Coa but does not interfere with the growth of *S. aureus*. Furthermore, we show through thermal shift and fluorescence quenching assays that isovitexin directly binds to Coa. Dynamic simulation and structure-activity relationship analyses suggest that V191 and P268 are key amino acid residues responsible for the binding of isovitexin to Coa. Taken together, these data indicate that isovitexin is a direct Coa inhibitor and a promising candidate for drug development against *S. aureus* infection.

## Introduction

*Staphylococcus aureus* is a major etiologic agent of community-associated and nosocomial infections. *S. aureus* infection can cause diseases ranging from minor soft tissue infection to more serious and life-threatening disorders, such as necrotizing pneumonia, endocarditis, septic shock syndrome, and sepsis [1]. The emergence and spread of antibiotic-resistant pathogens has greatly compromised therapeutic alternatives and increased morbidity and mortality in hospitalized and susceptible patients [2]. The declining effectiveness of conventional antibiotics and the lag in the development of new antimicrobial drugs pose a serious threat to public health worldwide. Therefore, the development of new therapeutics to control *S. aureus* infection is urgently needed [3].

*S. aureus* exploits diverse virulence factors to colonize and subsequently destroy host cells and tissues while protecting itself from the host's immune system. In essence, these virulence factors are the main causes of persistent infections and clinical symptoms such as abscesses, inflammation, and sepsis [4]. Various preclinical experiments have confirmed that targeting virulence factors can significantly attenuate infection, and the development of therapeutics that targets bacterial virulence rather than survival is a promising strategy to control *S. aureus* infection [5].

Plasma clotting by *S. aureus* may be the earliest recognized phenomenon of bacterial virulence. *S. aureus* can induce blood clotting by two prothrombin-activating proteins, coagulase (Coa) and von Willebrand factor-binding protein (vWbp) [6, 7]. Coa inserts its N-terminal peptide sequence into the activation pocket of prethrombin-2 and activates the host hemostatic factor by allosterically inducing the functional catalytic machinery. The Coa-prothrombin complex, also known as staphylothrombin, can convert fibrinogen to insoluble fibrin [8, 9]. Then, transglutaminase fibrin-stabilizing factor (FXIII) crosslinks the fibrin cables to form polymerized fibrin [10]. *S. aureus* clumping factor A (ClfA) can bind to the polymerized fibrin, thus agglutinating *S. aureus* into large bacterial aggregates surrounded by a fibrin meshwork. This causes abscesses, thromboembolic lesions, and even lethal sepsis [11]. Experiments in various animal models have shown that staphylothrombin-mediated fibrin generation not only enhances *S. aureus* virulence but also enables *S. aureus* to form a pseudocapsule and evade opsonophagocytic clearance by the host innate immune system [12-14]. Furthermore, *S. aureus*-induced coagulation causes thromboembolic events that contribute to bacterial spread to other organ systems. Staphylothrombin-generated fibrin facilitates bacteria-platelet interactions and contributes to the activation of platelets [15].

Due to this nonenzymatic activation of prothrombin, staphylothrombin is not inhibited by most anticoagulant drugs, such as heparins, ethylenediaminetetraacetic acid (EDTA), or sodium citrate [7, 16, 17]. However, two small molecule inhibitors of staphylothrombin, dabigatran and argatroban, have been developed [18, 19], and they inhibit staphylothrombin-mediated coagulation by targeting the catalytic site of thrombin. Although dabigatran and argatroban can prevent excessive coagulation caused by *S. aureus*, inhibition of host thrombin poses a bleeding risk to patients in clinical practice [20]. The importance of *S. aureus* Coa as a virulence factor has been recognized for decades, but there is a lack of effective inhibitors for this enzyme. Direct inhibitors of *S. aureus* Coa that do not affect host hemostasis would provide a useful therapeutic alternative to alleviate the excessive coagulation caused by *S. aureus*.

**Fig. 1:**
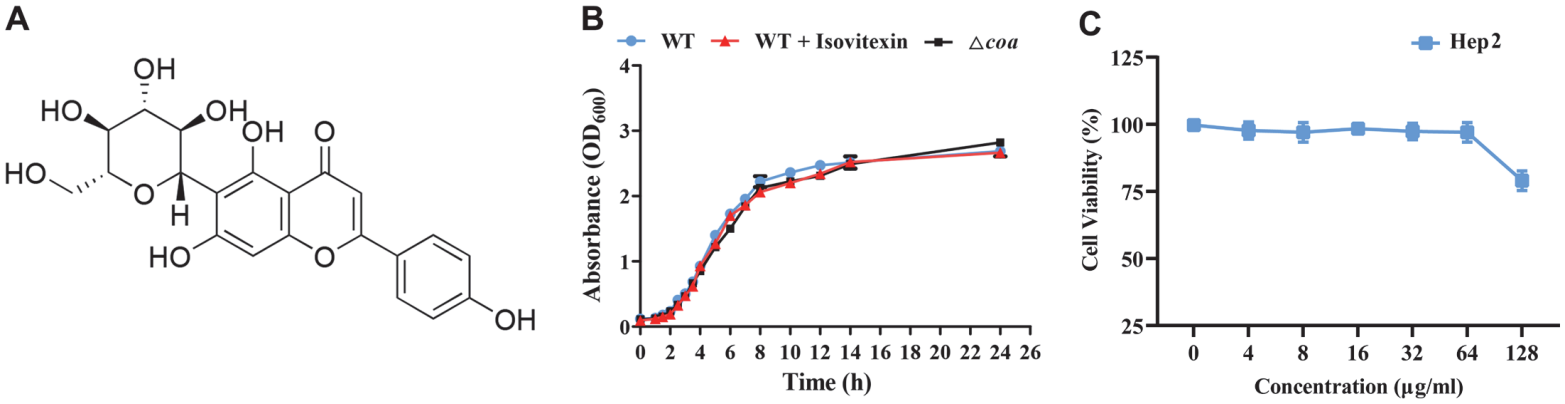
Isovitexin did not affect the growth of *S. aureus*. (**A**) Structure of isovitexin. (**B**) Growth curve of *S. aureus* Newman treated with or without isovitexin (256 μg/ml). Δ*coa* served as control. (**C**) Cytotoxicity of isovitexin (32, 64, and 128 μg/ml) on HEp-2 cells.

Compounds extracted from medicinal plants are a rich source of unique phytochemicals, as many novel bioactive products from traditional Chinese medicine have been shown to display significant anti-virulence activity [21-23]. Isovitexin (apigenin-6-C-β-glucopyranoside) (Fig. 1A) is a mono-C-glycosylflavone with one sugar moiety attached to the flavone skeleton, and has been found in diverse natural plant sources. It has been shown to have various therapeutic properties, such as antioxidant, anti-inflammatory, and anti-Alzheimer’s disease (AD) activities [24]. In this study, we found that isovitexin prevents excessive coagulation caused by *S. aureus* and is a direct inhibitor of *S. aureus* Coa. Therefore, isovitexin is a promising lead compound for further development as a therapeutic agent against *S. aureus* infection.

## Materials and Methods

### Bacterial Strains, Culture Conditions and Reagents

*S. aureus* Newman strain D2C (American Type Culture Collection) and its mutant Δ*coa* strain were used throughout this study. *S. aureus* was propagated in brain heart infusion (BHI) broth or on BHI agar plates at 37°C. *Escherichia coli* (*E. coli*) was propagated in Luria-Bertani (LB) broth at 37°C. *E. coli* strain BL21 (Novagen) containing pET15b-Coa was used to overexpress the Coa protein [23] and was routinely cultured in LB broth supplemented with ampicillin (100 μg/ml). Isovitexin and all reagents were purchased from Sigma-Aldrich (China). Isovitexin was dissolved and stored in dimethyl sulfoxide (DMSO) at 4°C.

### Determination of the *S. aureus* Newman Growth Curve

Ten microliters of preserved *S. aureus* Newman and its knockout strain Δ*coa* were inoculated into 3 ml of BHI medium and cultured overnight (200 r/min, 37°C). The overnight-cultured *S. aureus* was inoculated at a 1:100 dilution into BHI medium with or without 256 μg/ml isovitexin, and the Δ*coa* strain was inoculated into normal BHI medium. The bacteria were cultured for 24 h (37°C, 200 r/min), and every 30 min, the OD_600_ was measured with an ultraviolet spectrophotometer.

### Cytotoxicity Assay

Cytotoxicity was determined using the Cell Counting Kit-8 (CCK-8) as previously described [25]. Briefly, HEp-2 cells (5 × 10^4^ cells/well) were seeded in a 96-well cell culture plate and then incubated at 37°C under 5% CO_2_ for 24 h. Subsequently, the medium was removed gently, and the freshly prepared medium containing various concentrations of isovitexin (0 to 128 μg/ml) or DMSO was added to the cells for an additional 24 h. Afterwards, CCK-8 solution (10 μl) was carefully added to each well and incubated for another 4 h in an incubator. The OD value at 450 nm was measured for assessing the cell viability.

### Site-Directed Mutagenesis and Preparation of Recombinant Coa

Purification of 6×His-Coa was carried out as described previously [14]. Briefly, *E. coli* strain BL21 (DE3) carrying pET15b-Coa was propagated in LB broth at 37°C. When the culture grew to the mid-log phase (an OD_600_ between 0.6 and 0.8), 0.5 mM isopropyl β-d-1-thiogalactopyranoside (IPTG) was added to the BHI broth to induce protein expression, and the bacteria were cultured for 12 h at 16°C. Cells were harvested, and Coa was purified using a 6 × His/Ni-NTA agarose column according to the manufacturer’s manual (GE Healthcare). The point mutants Y188A, V191A, N267A and P268A were generated from pET15b-Coa using a site-directed mutagenesis kit (TransGen Biotech). The primers used for site-directed mutagenesis are listed in Table 1.

### Coagulation Assay for Coa Inhibition

The tube coagulation test was performed by mixing 5 μl of Coa protein (100 μM) with 195 μl of fresh rabbit blood containing different concentrations of isovitexin (0, 64, and 128 μg/ml). The tubes were incubated at 37°C. The level of blood coagulation was evaluated by tilting the tubes at 45° angles every 10 min. The test was considered positive if the rabbit blood formed a visible clot.

The plate coagulation assay was performed according to the experimental method of Hwang *et al*. [26]. In brief, 0.4% poly (ethylene glycol) (PEG) 8000, 3 mg/ml fibrinogen, 1% fresh anti-condensed rabbit plasma, and 0.9%agarose were mixed in a sterile Erlenmeyer flask, and the mixture was poured into a 60-mm cell culture plate. Five holes were punched in the agarose plates. Twenty microliters of Coa at different concentrations (5 mg/ml, 2.5 mg/ml, 1.25 mg/ml, 0.625 mg/ml, and 0.3125 mg/ml) was added to the wells. According to the size of fibrin precipitation, 18 μl of protein (at the indicated concentration) was mixed with 2 μl of isovitexin (final concentration 0, 16, 32, 64, or 128 μg/ml) and added to the wells. The plates were incubated overnight at 37°C, and the inhibitory activity of isovitexin against Coa was determined by the size of the coagulation region.

### Fluorescence-Based Thermal Shift Assay

The fluorescence-based thermal shift assay was used to investigate the binding of isovitexin to Coa and was performed as previously described [27]. A sensitive fluorescent dye, SYPRO Orange (Invitrogen), was used to monitor the thermal stability of Coa. First, 5000× SYPRO Orange was diluted 50-fold with buffer solution (150 mM NaCl and 10 mM HEPES, pH 7.5) before mixing with the protein. One microliter of 100× SYPRO Orange, 2 μg of Coa, 2 μl of isovitexin (64 μg), and the appropriate volume of buffer were then added to PCR tubes for a total volume of 20 μl. Tubes were spun at 200 ×*g* for 1 min to remove bubbles from the solution. The PCR tubes were then put into an IQ5 Real-Time PCR Detection System (Bio-Rad) and heated from 25 to 95°C at a rate of 1°C/min. The fluorescent signal was measured every 10 s with Ex/Em: 490 nm/530 nm. These experiments were performed twice with three replicates per sample. The data were analyzed with Bio-Rad iQ5 software and Origin 8.0 software as described previously [28].

### Fluorescence Quenching Assay

To determine the binding affinity of isovitexin to Coa, the *K_A_* value of isovitexin to Coa was measured with a fluorescence quenching assay as previously described [29]. Briefly, different amounts of isovitexin, ranging from 0 nM to 57.9 nM, were mixed with buffer (100 mM NaCl and 10 mM Tris [pH 7.4]) containing 4 μM purified Coa. A fluorescence spectrophotometer (RF5301, Japan) was used to measure the fluorescence emission spectra (290 –500 nm). The *K_A_* value was calculated using SPSS (version 20.0). These measurements were performed in triplicate.

### Molecular Docking and Dynamics

Molecular docking and dynamics were used to study the binding mode of isovitexin and *S. aureus* Coa. The three-dimensional (3D) structure of the Coa was predicted by SWISS-MODEL, a fully automated protein structure homology modeling server. The 3D structure of isovitexin was drawn using both ChemBioDraw Ultra 14.0 and ChemBio3D Ultra 14.0 software. The AutoDockTools 1.5.6 package [30, 31] was used to generate the docking input files. The Amber 14 [32-34] and AmberTools 15 programs were used for molecular docking and dynamics simulations of the selected docked pose, and molecular dynamics simulations were carried out for 40 ns. All molecular dynamics simulations were performed on a Dell Precision T5500 workstation. The binding free energy (Δ*G_bind_*) was calculated by the Molecular Mechanics/Generalized Born Surface Area (MM/GBSA) method using AmberTools 15. Moreover, to study the key protein residues responsible for ligand binding, the binding free energy was decomposed on a per-residue basis. For each complex, the binding free energy of MM/GBSA was estimated as follows: Δ*G_bind_* = *G_complex_* ‒ *G_protein_* ‒ *G_ligand_*, where Δ*G_bind_* is the binding free energy and *G_complex_*, *G_protein_*, and *G_ligand_* are the free energies of the complex, protein, and ligand, respectively.

## Results

### Growth Curve of *S. aureus* and Cytotoxicity of Isovitexin

To determine whether isovitexin affects the growth of *S. aureus*, we measured the growth of *S. aureus* Newman in the presence of various concentrations of isovitexin. The growth curve shows that in the presence of 256 μg/ml isovitexin, *S. aureus* growth was not affected. Additionally, we observed no difference in the growth rates of the wild-type (WT) and mutant Δ*coa* strains in the absence of drug (Fig. 1B). Therefore, 256 μg/ml isovitexin had no inhibitory effect on the growth of *S. aureus* Newman in vitro. In addition, the CCK-8 assay was subsequently used to evaluate the cytotoxicity of isovitexin in HEp-2 cells with the results showing that 64 μg/ml isovitexin had no cytotoxicity on the HEp-2 cells (Fig. 1C).

### Isovitexin Inhibits *S. aureus* Coa Activity

**Fig. 2:**
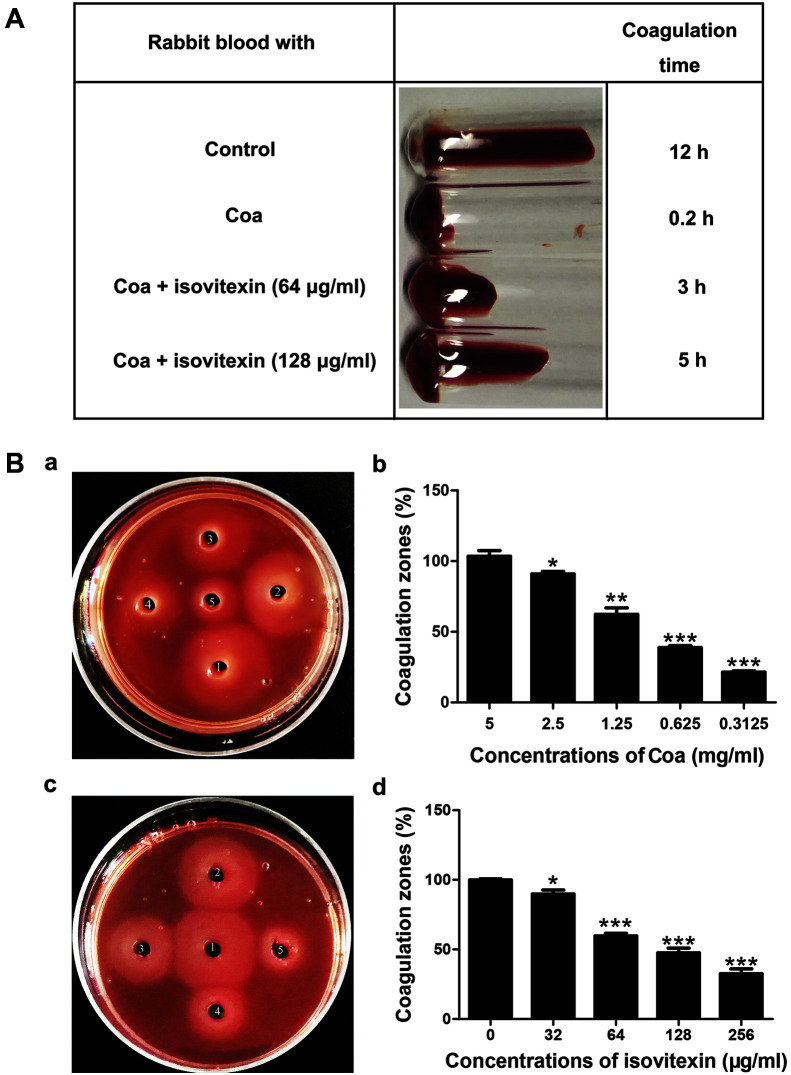
Isovitexin had an inhibitory effect on the coagulation activity of Coa. (**A**) Effect of various concentrations of isovitexin (0, 64, and 128 μg/ml) on the coagulation time of Coa. (**B**) a, Coagulation activity of various concentrations of Coa (from well 1 to 5 are 5, 2.5, 1.25, 0.625, and 0.3125 mg/ml, respectively); c, 2.5 mg/ml Coa was mixed with various concentrations of isovitexin (0, 32, 64, 128, and 256 μg/ml) and added to wells 1 to 5; b, d, the sizes of the coagulation zones. *p < 0.05, **p < 0.01 and ***p < 0.001 were calculated using one-way ANOVA.

To investigate the inhibitory effects of isovitexin on the coagulation activity of Coa, a tube coagulation assay was performed. The results showed that coagulation time increased with increasing concentrations of isovitexin (Fig. 2A), indicating that isovitexin inhibits Coa activity in a dose-dependent manner. To further study the clotting ability of Coa, a plate coagulation assay was performed. In the absence of drug, fibrinogen could be converted to insoluble fibrin by Coa, forming obvious turbid halos on the agar plate. The size of the halo indicates the strength of the enzymatic activity. First, the appropriate protein concentration for the plate coagulation experiment was determined. As shown in Fig. 2B-a, when the protein concentration increased from 0.3125 mg/ml to 5 mg/ml, the size of the coagulation zone increased. Compared with well 1, the sizes of the coagulation zones in wells 2-5 were 90.93%, 62.35%, 38.78%, and 21.43%, respectively (Fig. 2B-b), indicating that Coa converts fibrinogen to fibrin in a dose-dependent manner. Based on the findings in Fig. 2B-a, 2.5 mg/ml Coa was selected for subsequent experiments. Different concentrations of isovitexin (0, 32, 64, 128, and 256 μg/ml) were added to wells 1-5, respectively (Fig. 2B-c). The diameters of the coagulation zones of wells 2–5 were 89.83%, 59.75%, 47.50%, and 32.54% of the diameter of well 1 (Figs. 2B-D). These results show that isovitexin significantly inhibits the activity of Coa at concentrations greater than 64 μg/ml.

### Isovitexin Directly Binds to Coa

**Fig. 3:**
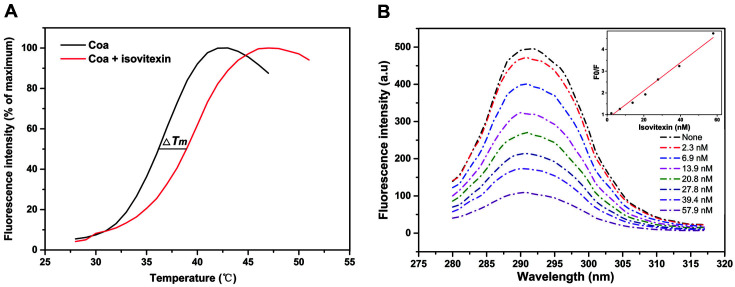
The interaction between Coa and isovitexin. (**A**) Isovitexin enhanced the thermal stability of the Coa protein. (**B**) Emission spectra of Coa in the presence of various concentrations of isovitexin at λex = 280 nm. Inset: Stern-Volmer plot depicting the fluorescence quenching caused by the combination of Coa and isovitexin.

We used a fluorescence-based thermal shift assay to further investigate the direct binding of isovitexin to Coa. When small molecules bind proteins, a conformational change in the protein is typically induced, which increases its thermal stability and results in an increase in melting temperature (*T_m_*). As shown in Fig. 3A, after incubation of 64 μg/ml isovitexin with Coa, the *T_m_* of Coa increased by 3°C, suggesting that isovitexin physically interacts with Coa in vitro.

The intrinsic protein fluorescence quenching assay is a basic method to measure ligand binding to proteins with high reliability and sensitivity [29]. We titrated 1 μM Coa with a final concentration of 0–57.9 nM (64 μg/ml) isovitexin and monitored the fluorescence emission with excitation at 280 nm. From these data, we determined the binding constant (*K_A_*) for isovitexin binding to Coa. As shown in Fig. 3B, as the concentration of isovitexin increased, the intrinsic fluorescence intensity of Coa decreased. The quenching constant of Coa by isovitexin was calculated by the Stern-Volmer equation to be significantly larger than 2.0 × 10^10^ L/mol·s, indicating that static quenching is the main mechanism. Using the static quenching formula Lg[(*F0-F*)/*F*] = LgK + nLgQ, the *K_A_* of isovitexin binding to Coa was determined to be 4.07 × 10^5^. This is further evidence that isovitexin directly binds to Coa.

### Binding Mode of Isovitexin with Coa

**Fig. 4:**
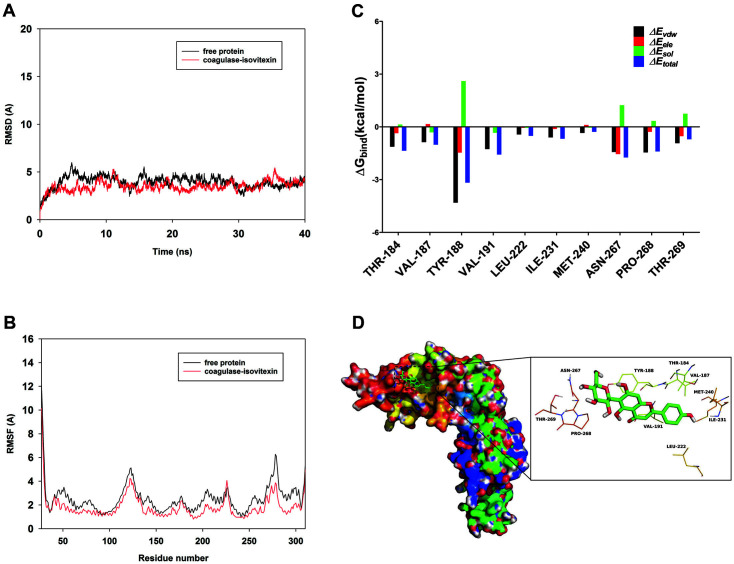
Binding mode of isovitexin with Coa. (**A**) Root-mean-square deviations (RMSDs) of all atoms of the Coaisovitexin complex. (**B**) RMSF of the residues of Coa in the free protein and the Coa-isovitexin complex. (**C**) The binding energy decomposition of the residues in the Coa-isovitexin complex. (**D**) The predicted binding pattern of isovitexin with Coa.

To explore the potential binding mode between isovitexin and Coa, we conducted molecular docking and molecular dynamics simulations. As shown in Fig. 4A, the protein structures of the two systems were stabilized during a 40-ns simulation. We then calculated the root mean square fluctuation (RMSF) of each amino acid residue to reveal its flexibility in the free Coa and in the Coa-isovitexin complex (Fig. 4B). Most of the residues at the Coa binding site had less flexibility in the Coa-isovitexin complex, and the RMSF values were less than 3 Å, indicating that these residues bind to isovitexin and have higher stability in the isovitexin-bound state. To study the residues around the binding site and their energy contributions to the system, calculations were carried out using the MM/GBSA method.

The summations of the per-residue interaction free energies were separated into Van der Waals (Δ*E_vdw_*), solvation (Δ*E_sol_*), electrostatic (Δ*E_ele_*), and total contributions (Δ*E_total_*). In the Coa-isovitexin complex, residue Tyr-188 had a weak electrostatic (Δ*E_ele_*) contribution, with a value of < –1.4 kcal/mol (Fig. 4C). Detailed analysis showed that residues Y188, V191, N267, and P268 with Δ*E_vdw_* of < –1.0 kcal/mol have appreciable Van der Waals interactions with isovitexin due to proximity. Aside from these residues, the majority of the decomposed energy interactions originated from Van der Waals interactions, apparently through hydrophobic interactions, such as those with Val-187, Leu-222, Ile-231, and Met-240 (Fig. 4D). The total binding free energy for the Coa-isovitexin complex was calculated according to the MM/GBSA approach, and the estimated Δ*G_bind_* of isovitexin to Coa was –17.6 kcal/mol. This suggests that isovitexin can strongly bind to and interact with the binding site of Coa. These possible interaction residues (Y188, V191, N267, and P268) are located in the D1 and D2 domain of Coa which are for prothrombin binding, so the binding of isovitexin may affect the formation of the staphylothrombin complex.

In summary, these molecular simulations provided a rational explanation for how isovitexin binds to Coa, which is valuable information for the further development of Coa inhibitors.

### Determination of the Binding Sites for Isovitexin on Coa

**Fig. 5:**
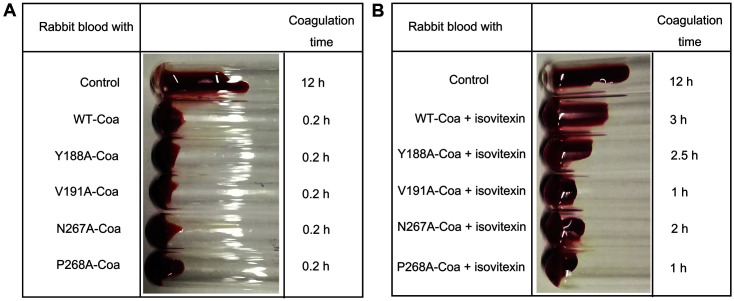
The coagulation activity of WT Coa and its mutant proteins in the (**A**) absence or (**B**) presence of isovitexin.

Based on predictions from our computational molecular docking and molecular dynamics simulations, we generated four Coa point mutants: Y188A-Coa, V191A-Coa, N267A-Coa, and P268A-Coa. We studied isovitexin binding to WT Coa and the point mutants using a fluorescence quenching assay and determined the *K_A_* values. We found that the point mutants had lower *K_A_* values when binding to isovitexin, with V191A-Coa and P268A-Coa showing the largest decrease in KA (Table 1). We also studied the effects of these point mutations on protein coagulation capacity. The coagulation activity of the point mutants was similar to that of WT Coa, indicating that these mutations do not affect the coagulation activity of Coa (Fig. 5A). However, the inhibitory effects of isovitexin on coagulation were significantly reduced for all mutants compared to the WT Coa protein. Mutation of V191 or P268 caused the most profound decrease in sensitivity to isovitexin (Fig. 5B). This suggests that the amino acid residues V191 and P268 are key sites for the interaction of isovitexin with Coa.

## Discussion

Multidrug-resistant *S. aureus* is a major bacterial causative agent of hospital and community-acquired pneumonia in adults and children, resulting in significant morbidity and mortality [2]. Therefore, there is an urgent need for novel and effective strategies to control *S. aureus* infection. Due to the clear importance of virulence factors in pathogenesis, antivirulence has been an area of heavy research interest [5].

Over 40 exotoxins are known to be associated with the success of *S. aureus* as a pathogen. In addition to these secreted toxins, *S. aureus* also produces two kinds of virulence factors: cofactors that activate host zymogens and enzymes for degradation of host tissue components [35, 36].

Due to its role in inducing coagulation of human plasma, Coa, a cofactor produced by *S. aureus*, has long been recognized as an important virulence factor and a promising pharmacological target for antivirulence strategies [17]. Coa can bind to prothrombin to form staphylothrombin, thereby inducing the cleavage of fibrinogen into fibrin, which forms a capsule encapsulating *S. aureus* and protects this bacterium from immune phagocytosis [14]. Previous studies have shown that *S. aureus* strains with Coa mutations exhibit attenuated virulence in mouse models of pneumonia, suggesting that Coa has a vital role in the pathogenesis of staphylococcal infections [23, 37].

Currently, two small molecules, dabigatran and argatroban, are used to treat *S. aureus* infection by inhibiting staphylothrombin [18, 19]. However, inhibition of thrombin by dabigatran and argatroban can cause bleeding, a serious side effect [20]. Therefore, inhibitors that can selectively inhibit Coa activity without affecting physiological thrombin activity would prove to be superior for clinical use.

Small molecules isolated from herbal medicines have structural diversity and good human tolerance that are difficult to match through chemical synthesis, and herbal small molecules may confer completely different mechanisms of action than chemically synthesized small molecule monomers. Currently, natural products are not only used for the direct treatment of diseases but are also used as lead compounds in the design of new drugs and have been regarded as an important source of drug development [38].

We systematically screened anti-Coa molecules from natural compounds via a blood coagulation assay and identified four natural molecules as specific inhibitors of Coa or vWbp [37-40]. Isovitexin (apigenin-6-C-glucoside), a natural flavonoid derived from Chinese herbs, possesses a variety of biological activities [24]. In this study, we found that isovitexin can directly bind to the Coa and inhibit its coagulation activity. This is the first report of the anticoagulation activity of this compound. To explore the mechanism of inhibition, we performed fluorescence-based thermal shift assays, intrinsic protein fluorescence quenching assays, molecular dynamics simulations, and site-specific mutagenesis.

In the thermal shift assay, incubation with isovitexin increased the *T_m_* value of Coa by 3°C. Using an intrinsic protein fluorescence quenching assay, we found that the KA of isovitexin binding to Coa was 4.07 × 10^5^. This evidence suggests a direct interaction between isovitexin and Coa. We then investigated the binding mechanism of Coa with isovitexin using molecular docking and molecular dynamics simulations and found that residues Y188, V191, N267, and P268 critically contribute to the formation of the stable Coa-isovitexin complex. We further confirmed this interaction using site-specific mutagenesis and fluorescence spectroscopy quenching analysis. The experimental data align closely with our theoretical calculations, and V191 and P268 are likely the key amino acids for the Coa-isovitexin interaction. In conclusion, this study demonstrates that isovitexin is a direct inhibitor of Coa.

## Figures and Tables

**Table 1:** The values of the binding constants (KA) based on the fluorescence quenching.

Proteins	WT-Coa	Y188A	V191A	N267A	P268A
KA (1 × 10^4^) L·mol^−1^	9.98	8.74	4.35	8.04	5.15

**Table 2:** Primers used in this study.

Primer name	Sequence (5’-3’)
Y188A-coa-F	AAGTAGCCGATCTCGTATCTGAAAT
Y188A-coa-R	CCTTAGTTGCTTTATCTTCTTCTGC
V191A-coa-F	TCTCGCATCTGAAATTGATACATTA
V191A-coa-R	TCGTATACTTCCTTAGTTGCTTTAT
N267A-coa-F	ATATGCTCCTACAACACATAACTAT
N267A-coa-R	TTCGTTATAGATTTCGGTCTATTTT
P268A-coa-F	TAATGCTACAACACATAACTATAAA
P268A-coa-R	TATTTCGTTATAGATTTCGGTCTAT
